# Feasibility of a guided participation discharge program for very preterm infants in a neonatal intensive care unit: a randomized controlled trial

**DOI:** 10.1186/s12887-019-1794-y

**Published:** 2019-11-04

**Authors:** S. Y. Lee, J. P. C. Chau, K. C. Choi, S. H. S. Lo

**Affiliations:** 1 Department of Pediatrics, Prince of Wales Hospital, 30-32 Ngan Shing Street, Shatin, N.T. Hong Kong SAR; 2 The Nethersole School of Nursing, Faculty of Medicine, The Chinese University of Hong Kong, Shatin, N.T. Hong Kong SAR

**Keywords:** Guided participation, Very preterm infants, Nurse-led, Discharge intervention, Mothers

## Abstract

**Background:**

Previous studies showed that parents of very preterm infants expressed feelings of incompetence and experienced high levels of stress upon the discharge of their infants. We conducted a systematic review of seven studies and observed potential benefits for parental outcomes when using discharge interventions that adopted guided participation (GP). More evidence is needed on the effective doses of discharge interventions underpinned by the principles of GP.

**Aim:**

To investigate the feasibility and preliminarily estimate the effects on parental competence and stress outcomes of a newly developed, nurse-led, GP discharge program for mothers of very preterm infants.

**Methods:**

A two-arm randomized controlled trial was conducted in a neonatal intensive care unit (NICU). Mothers of infants with gestational ages of ≤32 weeks who had no congenital malformations and did not need to undergo major surgeries were recruited. All mothers were the primary caregivers to their infants. The intervention group received a nurse-led GP discharge intervention (three structured 30- to 60-min GP sessions and one follow-up phone call). The control group received usual care. The parental outcomes were measured using the Parenting Sense of Competence Scale (C-PSOC) and Perceived Stress Scale (C-PSS) at baseline (T0), on the day of discharge (T1), after the follow-up phone call (within 72 h after discharge) (T2), and 1 month after discharge (T3). The outcomes were analyzed using generalized estimating equations based on intention-to-treat principles.

**Results:**

Thirty infant–mother dyads were recruited. Greater improvements in the C-PSOC score were observed in the intervention group than in the control group at T1 and T2, although these differences were statistically insignificant. The intervention group exhibited greater improvements than the control group in the C-PSS scores at T1, T2, and T3, although these differences were also not statistically significant.

**Conclusions:**

The findings suggest that a GP discharge intervention could improve parenting competence and stress among mothers with very preterm infants. The absence of adverse events suggests that the GP discharge intervention could be feasibly implemented in NICU settings. This feasibility study was not powered to determine the effectiveness of the intervention but is anticipated to lay the foundation for a future full-scale study.

**Trial registration:**

ClinicalTrials.gov Identifier: NCT03668912. Date of registration: 13 September 2018 (retrospectively registered).

## Background

Parents face various challenges while caring for very preterm (VP) infants, given the spectrum of health problems consequent to preterm births [1, 2]. Several studies have reported that parents of VP infants experience high levels of stress and express feelings of incompetence upon the discharge of their infants from the hospital [3–5]. In an exploration of the transitions experienced by parents caring for VP infants, Boykova and Kenner [6] reported that the parents were frightened or felt stressed by the environment of the neonatal intensive care unit (NICU), subsequent health issues, parenting failures, and helplessness after discharge. Another study reported that mothers of VP infants experienced distress and that having a VP infant had a major impact on their parenting self-image and their early bond with the infant [7]. The high parenting stress experienced by parents of VP infants not only caused issues with their health, such as anxiety or depression, but also difficulties in exerting their parenting role [8]. Common discharge interventions provided to parents of VP infants (birth weight < 1500 g) include educational programs enabling parents to read cues from infants, empowerment programs to strengthen parental competence, behavioral training to enhance infants’ cognitive development, home visits to assess parental competency, and follow-up phone calls to clarify health management plans [9–12].

We previously conducted a systematic review of seven studies to examine the effects of discharge interventions on the outcomes for parents of VP infants [13]. However, the findings were inconclusive, given the diverse foci and scopes of the included studies. Nonetheless, three of the included studies suggested the potential benefits of discharge interventions, specifically those using guided participation (GP) to coach parents in infant care, on the parents’ levels of sense of competency and perceived stress [13]. GP is a process through which an experienced person helps an inexperienced person to become competent in practice. This process offers a method for coaching novice parents in the care of their VP infants [14–16]. However, more evidence is needed regarding the effective doses of interventions underpinned by the principles of GP.

## Methods

### Aim

This study aimed to determine the feasibility and potential effectiveness of a newly developed, nurse-led GP discharge intervention in terms of the effects on the parenting sense of competency (PSOC) and perceived stress of mothers with VP infants. Assessment of feasibility of the intervention included the ease of recruitment and the adverse events occurring that were related to the implementation of the intervention. The results will help to justify a full-scale randomized controlled trial (RCT) to determine the effects of the GP discharge intervention.

### Design

This was a single-centered, 2-parallel-arm randomized controlled trial.

### Setting

The study was conducted in the Level III NICU of a regional teaching hospital that provides neonatal intensive care and specialized baby care services to a population of 1.5 million. The hospital averages 6000–7000 deliveries, including 100 VP infants, per annum.

### Participants

All infants admitted to the NICU were recruited during the study period if they met the following criteria: (1) a gestational age of ≤32 weeks at birth, (2) no congenital malformations, and (3) no need to undergo major surgery. The infants’ mothers were included if they (1) were the infant’s primary caregiver and (2) had no mental illness diagnosed by a physician at the time of recruitment. Eligible dyads of an infant and his/her parent were recruited. Lancaster, Dodd, and Williamson suggested that a sample size of at least 30 participants was adequate for a pilot study [17]. A sample size of at least 30 participants (dyads) was therefore deemed adequate for this feasibility study.

### Intervention

Dyads randomly allocated to the intervention group received usual care plus the GP discharge intervention, which was delivered by the same nurse consultant (NC) specializing in neonatal care. The major role of an NC in Hong Kong is to lead development of nursing practice within a specialty area. The intervention comprised three structured GP sessions and one follow-up phone call. GP sessions were offered at 33–34 weeks postconceptional age (PCA), 34–35 weeks PCA, and 48 h before hospital discharge. Each GP session was provided immediately after the usual updates on the infant’s condition and each session lasted for 30–60 min, depending on the severity of the infant’s condition and the mother’s understanding of the information provided, concerns, and performance in a return demonstration [see Additional file 1 for an outline of the GP sessions]. The contents of the GP sessions were developed with reference to the NICU discharge module endorsed by the National Association of Neonatal Nurses (2013) [18]. The follow-up phone call was provided within 72 h of the infant’s discharge from the hospital to provide mothers with information about community health care support and address their possible concerns about infant care.

An expert panel of six healthcare professionals specializing in pediatric care (two NCs, two registered nurses, and two physicians) and two mothers of VP infants validated the content of the program. Six pilot sessions involving the mothers of two infants were conducted. The program was delivered according to the intervention protocol, and no changes were made.

### Usual care

The usual care received by the control group included three usual condition updates at 33–34 weeks PCA, 34–35 weeks PCA, and 48 h before hospital discharge, as well as one follow-up phone call within 72 h after the infant’s discharge. The contents of the update included a brief update given to the mothers about the infants’ current clinical condition, feeding frequency, feeding volume, and vaccinations given. The usual practice also included repeated condition updates throughout hospitalization. The condition updates and follow-up phone call were conducted by either the NC or a physician of the NICU.

### Outcome measures

The primary outcome was the mothers’ efficacy and satisfaction with parenting as assessed by the Chinese version of the PSOC Scale (C-PSOC) [19, 20]. The C-PSOC includes 17 items on two subscales (efficacy and satisfaction). Each item was rated on a 6-point Likert scale from 1 “Strongly disagree” to 6 “Strongly agree”. The total score was yielded by summing all item scores and ranged from 17 to 102. A higher score indicates a higher sense of competence and satisfaction. The scores showed a Cronbach’s alpha of 0.85 and acceptable construct validity.

The secondary outcome was the mothers’ level of perceived stress as assessed by the Chinese version of the 10-item Perceived Stress Scale (C-PSS) [21, 22]. PSS is a widely used psychological instrument for assessing the extent to which a person’s life events are perceived as stressful. Each item was rated on a 5-point Likert scale from 0 “Never” to 4 “Very often”. All item scores were summed to give a total score from 0 to 40. The higher the score was, the greater the perceived stress was. The C-PSS showed a Cronbach’s alpha of 0.80 and acceptable construct validity.

Adverse events were determined by the mothers’ verbalization of negative feelings or discomfort after receipt of the intervention. Ease of recruitment was determined by the numbers of participants who were approached, who consented, and who remained in the follow-up measurements.

### Data collection

Ethical approval was obtained from the Joint Chinese University of Hong Kong–New Territories East Cluster Clinical Research Ethics Committee. VP infants and their mothers were consecutively screened for eligibility by the NC after their admission to the NICU. A physician at the NICU conducted an initial interview and assessment with the mothers. The contents of the interview included explanation of the infant’s condition, the procedures performed, the management plan, potential complications of VP infants, and problems facing parents within 72 h after delivery of a VP infant. After that, all eligible mothers were asked to provide written informed consent before the collection of baseline data. Next, the mothers were randomly assigned to the intervention or control group according to a computer-generated sequence of random numbers. Concealed allocation was performed using sequentially numbered and sealed envelopes. Data were collected at baseline (within 72 h of admission to the NICU) (T0), on the day of discharge (T1), after a follow-up phone call (within 72 h after hospital discharge) (T2), and during a follow-up appointment (1 month after discharge) (T3). The data collector was blinded to intervention allocation.

### Statistical analysis

The normality of continuous data was assessed using skewness and kurtosis statistics and a normal probability plot. The outcomes were analyzed using generalized estimating equations (GEE) on the basis of intention-to-treat principles. Differential changes in each outcome at T1, T2, and T3 relative to the baseline, T0, were compared between the two groups using the time and group interaction terms in the underlying GEE model of the outcome. As the infants’ birth weights and gestational ages were non-comparable between the control and intervention groups, these two factors may have been potential confounding factors affecting the responses of the parental outcomes. Accordingly, these factors were adjusted in the GEE analysis of the outcomes to improve the precision of the effect estimates. All statistical analyses were conducted using IBM SPSS 24.0 (IBM Corp., Armonk, NY, USA). All statistical tests were two-sided at a 5% level of significance.

## Results

**Fig. 1:**
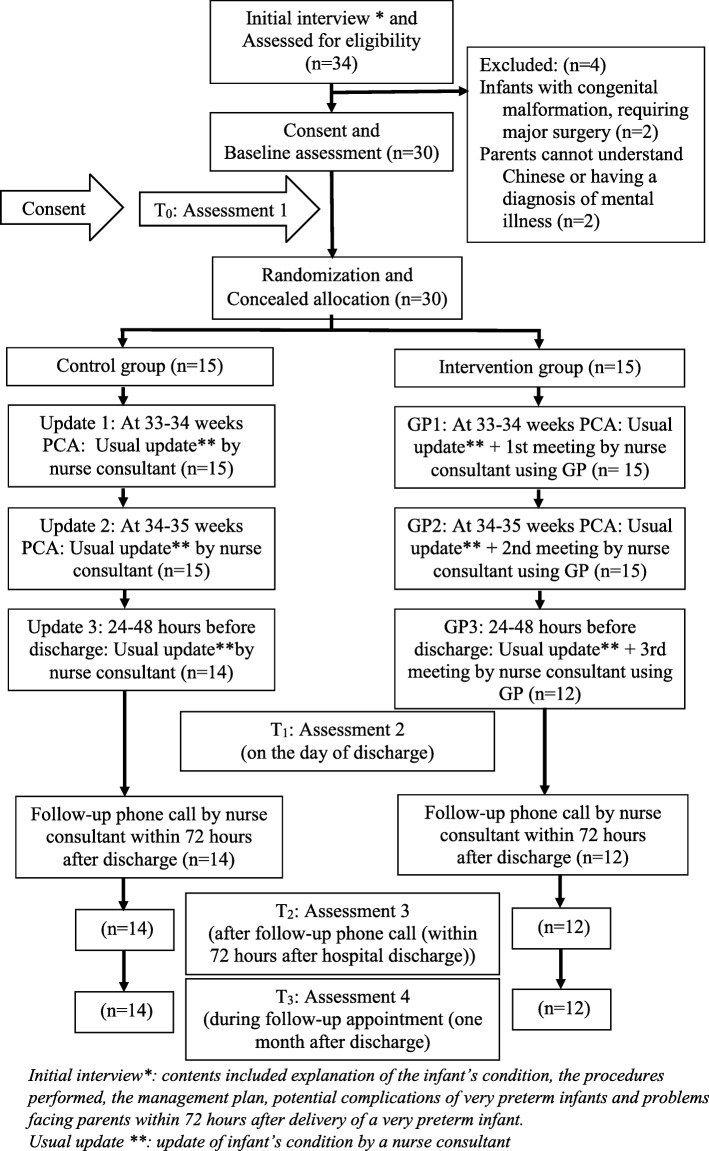
The study flow chart

A total of 30 infant–mother dyads (15 dyads per group) were recruited between March and October 2016 (Fig. 1). All of the mothers were the primary caregivers of the infants (age range: 23–43 years), and most had completed secondary education (Table 1). The infants were distributed evenly with respect to sex, and the characteristics of the two groups were comparable except for the significantly lower average birth weights and gestational ages of infants in the intervention group. One participant in the control group was discharged early as recommended by the physician, and that participant’s data could not be collected at T1, T2, and T3. Three participants in the intervention group could not undergo the assessments at T1, T2, and T3 during the trial period because of long hospital stays.

**Table 1:** Baseline characteristics of the dyad participants (*N* = 30)

Characteristics	Control (*n* = 15)	Intervention (*n* = 15)
Primary carers		
Age (years) ^†^ [range: 23–43]	33.40 (3.74)	32.80 (4.60)
Female gender	15 (100%)	15 (100%)
Relationship with the infant participant		
Mother	15 (100%)	15 (100%)
Previous loss of pregnancy or a child		
No	9 (60.0%)	7 (46.7%)
Yes	6 (40.0%)	8 (53.3%)
Educational level		
Secondary school or below	9 (60.0%)	10 (66.7%)
University or above	6 (40.0%)	5 (33.3%)
Employment status		
Housewife	9 (60.0%)	8 (53.3%)
Employed	6 (40.0%)	7 (46.7%)
Number of adults living together		
≤ 2	10 (66.7%)	12 (80.0%)
> 2	5 (33.3%)	3 (20.0%)
Need to look after other children at the same time		
No	6 (40.0%)	6 (40.0%)
Yes	9 (60.0%)	9 (60.0%)
Domestic helper at home		
No	10 (66.7%)	12 (80.0%)
Yes	5 (33.3%)	3 (20.0%)
Infants		
Gestational age at birth (weeks) ^†^ [range: 23.71–31.86]	29.70 (1.57)	27.46 (2.71)*
Birth weight (g) ^†^ [range: 596–1890]	1337.73 (302.43)	1057.40 (416.60)*
Singleton or multiple birth		
Singleton	12 (80.0%)	15 (100%)
Multiple birth	3 (20.0%)	0
Gender		
Female	6 (40.0%)	6 (40.0%)
Male	9 (60.0%)	9 (60.0%)
Mode of delivery		
Normal spontaneous delivery	8 (53.3%)	10 (66.7%)
Others	7 (46.7%)	5 (33.3%)
Any complications^		
No	6 (40.0%)	5 (33.3%)
Yes	9 (60.0%)	10 (66.7%)

Data marked with ^†^ are presented as mean (standard deviation), all others are presented as frequency (%)

*Significantly lower than the control group in *p* < 0.05 level

^The complications included respiratory distress, feeding intolerance, and necrotising enterocolitis, patent ductus arteriosus, retinopathy of prematurity and infection

Table 2 presents the results of the outcomes across time. The crude and adjusted GEE models for the C-PSOC and C-PSS scores (measuring mothers’ efficacy and satisfaction with parenting, and level of perceived stress respectively) yielded essentially similar results, which are presented on the basis of the adjusted model in Table 3. The mean baseline C-PSOC score of the intervention group was slightly but not significantly higher than that of the control group (regression coefficient, B = 1.2; *p* = 0.80) after adjusting for birth weight and gestational age. Both groups exhibited significant improvements in the C-PSOC score at T1, T2, and T3 relative to T0. Although the improvements in the C-PSOC scores of the intervention group at T1 and T2 were greater than those of the control group, these differences did not reach statistical significance [B for the group by time interaction = 0.27 (*p* = 0.92) and 0.57 (*p* = 0.89) at T1 and T2, respectively]. At T3, however, the improvement in the intervention group was smaller than that in the control group, although the difference was also not statistically significant (B for the group by time interaction at T3 = 0.9; *p* = 0.82).

**Table 2:** Parenting sense of competence and perceived stress level across time

Outcomes	Control (*n* = 15)	Intervention (*n* = 15)
Parenting sense of competence [Possible range: 17–102]		
T_0_	67.9 (12.0)	67.1 (11.4)
T_1_	73.6 (10.4)	71.3 (11.7)
T_2_	73.0 (10.1)	71.6 (9.2)
T_3_	73.6 (9.8)	72.1 (12.4)
Perceived stress level [Possible range: 0–40]		
T_0_	19.5 (5.3)	18.5 (5.5)
T_1_	17.5 (5.2)	15.7 (5.3)
T_2_	17.1 (4.6)	15.4 (3.0)
T_3_	15.3 (4.7)	14.2 (4.5)

All data are presented as mean (standard deviation)

**Table 3:** Generalized estimating equation models for comparing the outcomes across time between the two groups

Outcomes	Regression coefficients of the GEE models			
	Crude model		Adjusted model #	
	B (95% CI)	P	B (95% CI)	*P*
PSOC				
Group	−0.80 (−8.87, 7.72)	0.84	1.20 (−8.20, 10.60)	0.80
T1	4.24 (0.87, 7.62)	0.14	4.16 (0.85, 7.48)	0.01
T2	4.17 (−0.37, 8.71)	0.07	4.00 (−0.40, 8.41)	0.07
T3	4.80 (−0.05, 9.60)	0.05	4.66 (0.08, 9.23)	0.05
Group* T1	0.35 (−4.58, 5.29)	0.89	0.27 (−4.64, 5.17)	0.92
Group* T2	0.57 (−7.16, 8.30)	0.88	0.57 (−7.07, 8.20)	0.89
Group* T3	- 0.75 (−8.66, 7.16)	0.85	−0.90 (−8.58, 6.78)	0.82
PSS				
Group	−0.93 (−4.67, 2.80)	0.62	−1.38 (−6.02, 3.26)	0.56
T1	−1.80 (−4.27, 0.68)	0.15	− 1.77 (− 4.24, 0.70)	0.16
T2	−2.23 (−5.33, 0.88)	0.16	−2.19 (−5.31, 0.92)	0.17
T3	−3.68 (− 6.99, − 0.37)	0.03	−3.61 (− 6.95, − 0.28)	0.03
Group* T1	−0.98 (− 4.87, 2.85)	0.62	−0.96 (− 4.83, 2.91)	0.63
Group* T2	− 0.88 (− 5.01, 3.24)	0.68	−0.81 (− 5.02, 3.39)	0.71
Group* T3	− 0.31 (− 4.46, 3.84)	0.88	−0.26 (− 4.49, 3.98)	0.91

*GEE* Generalized estimating equations

Only the model estimates of regression coefficients of the dummy variables for the group [Group: 0 = Control (reference); 1 = Intervention], time points (T1, T2 and T3 with the baseline (T0) as reference), time points and group interaction terms (Group*T1, Group*T2 and Group*T3) are shown for the generalized estimating equation models

^#^With adjustment for birth weight and gestational age of the very preterm infants

After adjusting for birth weight and gestational age, the mean baseline C-PSS score (measuring mothers’ level of perceived stress) in the intervention group was slightly but not significantly lower than that in the control group (B = − 1.38; *p* = 0.56). Both groups exhibited improvements in the C-PSS scores at T1, T2, and T3 relative to T0. The intervention group exhibited even greater improvements than the control group at T1, T2, and T3 (B for group by time interaction = − 0.96 (*p* = 0.63), − 0.81 (*p* = 0.71), and − 0.26 (*p* = 0.91), respectively), but these differences did not reach statistical significance.

No adverse events were reported related to the implementation of the program. No verbalization of negative feelings or discomfort was reported by the mothers after receiving the intervention.

A total of 30 mothers were approached during the recruitment period and all of them agreed to participate in the study. Only four participants were lost to follow up at T1, T2, and T3 due to a condition unrelated to the program (early discharge or longer hospital stay than the study period).

## Discussion

This study was the first to examine the feasibility and potential effects of a GP discharge intervention program on parental outcomes among Chinese mothers with VP infants. A total of 30 infant–mother dyads were recruited in this study. The intervention and control groups were comparable except that the infants of the intervention group had significantly lower average birth weights and gestational ages. These two variables were adjusted in the outcome analysis using the GEE model. This study conducted simple randomization of the intervention and control groups at a 1:1 ratio. It would be helpful in future studies to conduct block randomization stratified in terms of ranges of birth weights and gestation ages to balance the distribution of this important characteristic in both the intervention and control groups.

VP infants are at higher risk of having more than one complication, which may affect the level of parental stress among parents [7, 8]. All infants recruited in this study had some form of neonatal complication. These included respiratory distress, feeding intolerance, necrotizing enterocolitis, patent ductus arteriosus, retinopathy of prematurity, and infection. However, there was no significant statistical difference in the number of neonatal comorbidities or complications between the intervention and control groups. Future studies with larger sample sizes could supply valuable data by accommodating additional confounders, including neonatal comorbidities or complications, in the adjusted outcome analysis.

### Potential effectiveness of the intervention

The mothers in this study expressed fair levels of their sense of competence in parenting and stress at baseline. These were comparable with previously published studies. One study found that mothers of preterm infants admitted to the NICU had fair levels of stress and maternal self-perceived competence regardless of experiencing moderately or severely preterm births [23]. Garfield et al. [24] reported that parents with very low birth weight infants experienced high levels of stress when bringing their infants home from the NICU. The level of stress was influenced by the parents’ perceived level of parenting competence.

In this study, although the improvements in the PSOC scores at T1 and T2 were greater in the intervention group than in the control group, this improvement was not sustained at T3. The results appear similar to those of studies in Western countries. Pridham et al. [25] evaluated the effects of GP on the feeding competencies of mothers with preterm infants. The results revealed significant improvements in the mothers’ regulation of negative affect and behaviors during feeding at one of four measurement time points during the first 12 post-term months. As the evaluation lasted for 12 months, the authors suggested that the focus of maternal concern might have shifted from nutrient intake to infant vulnerability and primary attentiveness.

Regarding perceived stress, the intervention group also exhibited greater but not statistically significant reductions in the perceived stress level relative to the control group at all time points. The mothers in this study encountered multiple difficulties in caring for the infants when the infants were initially discharged from hospital, which caused high levels of maternal stress. However, even without the provision of GP discharge intervention, the mothers might have realized that they could manage the situation after an adaptation period. Therefore, the perceived stress level in the intervention group decreased gradually to a level that was sustained until T3.

### Intervention component

The participating parents in both the intervention and control groups were provided with a follow-up phone call within 3 days of the discharge of their VP infants. The purpose of this call was to promptly address the mothers’ possible concerns about infant care. Additionally, the calls made to the participants (mothers) in the intervention group aimed to ensure the mothers’ connections with community resources such as private medical practitioners, maternal child health centers, and nearby emergency departments. In a review, Jefferies [26] observed that parents of VP infants often did not feel physically and psychologically well-prepared to care for their infants at home. Therefore, it was deemed important to advocate for community resources and thus ensure the safe discharge of high-risk infants. However, only one follow-up phone call was made by the NC 3 days after the infants’ discharge, which might not have been sufficient to address the mothers’ queries promptly. Therefore, alternatives such as a telephone hotline or telephone-based enquiry support during the initial 3 days after hospital discharge may be considered to provide more prompt support to mothers during the transition period.

The GP discharge intervention consisted of three 30–60-min sessions before the infants were discharged from the hospital. However, the dose of the intervention may not have been adequate to significantly improve the perceived efficacy and satisfaction of parenting competency among the parents who experienced VP births with various and demanding health needs. A study of 183 children at < 30 weeks’ gestation reported that parenting stress and family functioning were affected for up to 7 years after the birth of the VP infants [27]. Confidence in parenting requires time to build up. It would be worthwhile to design interventions providing more sessions or support after discharge, and to design a study with a longer follow-up period to capture the possible changes in the outcomes.

### Limitations

This study had some limitations of note. First, it was conducted in a single NICU, which affects the potential generalizability of the findings to all mothers with VP infants. Second, the participating mothers in both the intervention and control groups might have been biased by possible contact and communication with each other about the interventions received. Third, no information was collected on significant events, such as infant re-hospitalization or family issues, that occurred during the study period. These events might have affected the outcomes. Fourth, the sample only included 30 mothers of VP infants in this feasibility study. The sample size was too small for adequate predictive power to generate statistically significant results on the outcomes. Sample sizes of 393, 64, and 26 participants per group are required to give 80% power to detect small, medium, and large effect sizes of 0.2, 0.5, and 0.8 respectively on the outcome variables at the 2-sided 5% level of significance. We anticipate, however, that the comparison of outcomes between the two groups may provide useful information on effect estimations for future research and meta-analysis. Fifth, no information on the mothers’ mental health and the VP infants’ health after the program was collected.

### Feasibility of a full-scale RCT

A full-scale RCT of this intervention would be feasible given some modifications. First, it was noted that the selected timing of the GP sessions might have been unsuitable. In this study, structured GP sessions were scheduled at 33–34 weeks and 34–35 weeks PCA, and at 48 h before hospital discharge. The contents of these sessions were designed according to the normal developmental milestones of preterm infants. For example, most infants begin to develop suckling and swallowing reflexes by 33–34 weeks after conception, at which time it should be appropriate to teach feeding [1]. However, previous studies reported that learning to feed a preterm infant can be a complicated and emotional experience in which the parents are required to learn specific techniques related to feeding and monitoring along with balancing the infants’ readiness to eat [15, 28]. Many health parameters must be observed and preterm infants may lag behind the progress of their peers. Therefore, the pre-arranged GP sessions might not have been administered at the ideal time for teaching feeding to mothers of VP infants. The infants’ physical development, rather than the PCA, may be a better basis on which to schedule the GP sessions.

In addition, individual infants differed in terms of progress, limitations, and physical condition, and therefore the sessions were delivered before some mothers were ready to receive instructions on, for example, feeding a preterm infant. Although all of the infants had reached 33–34 weeks PCA, some remained in a critical condition and required aggressive ventilatory support due to frequent repeated desaturation; accordingly, only minimal handling of these infants was allowed [29]. Therefore, the NC in this study tailored the GP sessions to the mothers’ and infants’ needs by explaining and demonstrating the use of tube-feeding and the methods required to change a diaper while maintaining the infants’ body temperature. Given the unstable physical conditions of the infants, cuddling might have been impossible, and extra time may have been needed to instruct the mothers in the performance of simpler tasks such as the gentle changing of diapers. Additionally, some mothers might have required more encouragement to perform these tasks. The GP sessions in this study lasted for 30–90 min. In future, flexibility may be needed when setting the durations of GP sessions.

Notably, no adverse events were reported throughout the study. The process of participant recruitment was smooth, in that all of the mothers who were approached were interested and consented to participate in the study. These results suggested that the program was relatively safe to implement and a full-scale RCT would be feasible.

### Implications for nursing research

Future studies could recruit infant and parent participants from the NICUs of different hospitals to improve the overall statistical power. Additionally, the effects on the parental outcomes of interventions offered at different time-points or among mothers with singletons, twins, or triplets could be explored using sub-group analyses. Furthermore, future studies could measure parental knowledge of the health problems of VP infants and the mothers’ mental health status, assess the VP infants’ health after intervention, conduct a process evaluation to determine the fidelity of the program, and conduct a survey of the mothers’ satisfaction with the nursing service in terms of enhancing competency in infant care.

## Conclusions

The findings suggest that a GP discharge intervention could improve parenting competence and perceived stress among mothers with VP infants. The absence of adverse events suggests that the GP discharge intervention could be feasibly implemented in NICU settings. This study was not able to determine the effectiveness of the intervention but is anticipated to provide information about its feasibility and preliminary estimates of its effects for planning a future full-scale study. Instead of using the PCA as a reference point, it might be better to consider the infants’ physical development when determining the best timing for the GP interventions.

## Supplementary information

**Additional file 1.** Outline of the content of the guided participation discharge intervention.

## Data Availability

The datasets generated and/or analyzed during the current study are not publicly available due to confidentiality agreement with participants but are available from the corresponding author on reasonable request.

## Abbreviations

C-PSOC: Chinese version of the PSOC Scale
C-PSS: Chinese version of the Perceived Stress Scale
GEE: Generalized estimating eqs.
GP: Guided participation
NC: Nurse consultant
NICU: Neonatal intensive care unit
PCA: Postconceptional age
PSOC: Parenting sense of competency
RCT: Randomized controlled trial
VP: Very preterm
