# Supplementary material for: Feasibility of a guided participation discharge program for very preterm infants in a neonatal intensive care unit: a randomized controlled trial
Source: BMC Pediatr. 2019 Nov 4;19:402. doi: 10.1186/s12887-019-1794-y (PMC6827218; doi:10.1186/s12887-019-1794-y)
Supplement: Supplementary file 1 — Additional file 1. Outline of the content of the guided participation discharge intervention. [file 12887_2019_1794_MOESM1_ESM.docx]

**Additional file 1: Outline of the content of the guided participation discharge intervention**

**First meeting:** 33 to 34 weeks post conception

Content:

1. Assess the carer’s understanding of the infant’s condition.
2. Update the carer the infant’s conditions including the diagnoses, possible complications associated with the conditions, management plan, and progress.
3. Answer all questions from the carer related to the progress of the infant.
4. Clarify any misunderstandings on the infant’s diagnoses and management plan.
5. Ask the carer whether he/ she has started preparing the infant’s discharge.
6. Discuss with the carer the necessities of preparation for the discharge.
7. Inform the carer the start of preparation of infant’s necessities at home e.g. an infant bed with clean linen, clean infant clothes, and milk bottles.
8. Demonstrate to the carer basic care including comforting the infant, changing diapers, feeding via a feeding tube or via a cup or bottle.
9. Encourage the carer to touch the infant and speak to the infant.
10. Praise the effort of the carer to try touching and caring for the infant.
11. Provide an opportunity for return demonstration. Observe and comment on any return demonstration by the carer.

**Second meeting**

Time of meeting: 34-35 weeks post conception

Content:

1. Assess carer’s understanding of the infant’s progress.
2. Update the carer the infant’s conditions including the diagnoses, possible complications associated with the conditions, management plan, and progress.
3. Answer all questions from the carer related to the progress of the infant.
4. Clarify any misunderstandings on the infant’s diagnosis and management plan.
5. Discuss with the carer the items he/ she has prepared for the discharge of the infant from the hospital.
6. Ask the carer whether he/ she has opportunities to feed the infant.
7. Assess the feeding of the infant by the carer and give advice whenever it is necessary. Praise for the good performance.
8. Initiate direct breastfeeding if it is the mode of choice.
9. Observe how the infant is taken out of bed and placed into the bed by the carer himself/ herself. Give advice whenever it is necessary. Praise for good performance.
10. Discuss with the carer the necessary preparation at home for infant feeding.
11. Discuss with the carer the best choice of feed for the infant and the supplement.
12. Ask the carer how to assess if the infant is having adequate nutrition.
13. Introduce the ways to monitor the growth of the infant.
14. Discuss with the carer the feeding progress of the infant.
15. Assess the carer’s knowledge regarding the possible presentation on feeding if the infant becomes sick.
16. Discuss with the carer how to care for the infant if there is vomiting or diarrhoea.
17. Discuss with the carer the normal feeding milestones.
18. Provide advice for the carer about when the infant should drink water and start semi-solid food.

**Third meeting**

Time of meeting: immediately before (24-48 hours) discharge

Content:

1. Assess the carer’s understanding of the infant’s progress.
2. Update the carer the infant’s conditions including the diagnoses, possible complications associated with the conditions, management plan, and progress.
3. Answer all questions from the carer related to the progress of the infant.
4. Clarify any misunderstanding on the infant’s diagnosis and management plan.
5. Ensure carer’s understanding of the medication regimens.
6. Demonstrate to the carer how the medication is administered. Provide an opportunity for return demonstration.
7. Discuss with the carer the continuation of breastfeeding.
8. Identify any concerns about protection and safety after the infant goes home, e.g. avoidance of overcrowding area, safety in bathing, infant’s bed, toys for small infants, smoking and environmental ventilation.
9. Discuss with the carer about the possible support networks, e.g. family, neighbours, and health care settings.
10. Introduce the carer to the health care settings for the infant including Accident and Emergency Department, local Maternal Child Health Centre, and general practitioners.
11. Assess carer’s knowledge about the vaccination program for the infant.
12. Introduce the immunization schedule and the vaccination record.

**Telephone follow up**

Time of phone call: within 72 hours after the very preterm infant is discharged from hospital

Content:

1. Discuss with the carer for any concerns related to infant care.
2. Address all concerns related to infant care and development.
3. Enquire whether the infant has received the services offered by the local Maternal Child Health Centre.
4. Emphasise the importance of attending follow up appointments at speciality out-patient clinic.
5. Suggest the carer to consult general practitioners if the infant gets sick.
